# CRISPR/Cas9-mediated mutagenesis of *VvbZIP36* promotes anthocyanin accumulation in grapevine (*Vitis vinifera*)

**DOI:** 10.1093/hr/uhac022

**Published:** 2022-02-20

**Authors:** Mingxing Tu, Jinghao Fang, Ruikang Zhao, Xingyu Liu, Wuchen Yin, Ya Wang, Xianhang Wang, Xiping Wang, Yulin Fang

**Affiliations:** College of Enology, College of Food Science and Engineering, Viti-viniculture Engineering Technology Center of State Forestry and Grassland Administration, Shaanxi Engineering Research Center for Viti-Viniculture, Heyang Viti-Viniculture Station, Northwest A&F University, Yangling, Shaanxi 712100, China; State Key Laboratory of Crop Stress Biology in Arid Areas, College of Horticulture, Northwest A&F University, Yangling, Shaanxi 712100, China; State Key Laboratory of Crop Stress Biology in Arid Areas, College of Horticulture, Northwest A&F University, Yangling, Shaanxi 712100, China; Key Laboratory of Horticultural Plant Biology and Germplasm Innovation in Northwest China, Ministry of Agriculture, Northwest A&F University, Yangling, Shaanxi 712100, China; College of Enology, College of Food Science and Engineering, Viti-viniculture Engineering Technology Center of State Forestry and Grassland Administration, Shaanxi Engineering Research Center for Viti-Viniculture, Heyang Viti-Viniculture Station, Northwest A&F University, Yangling, Shaanxi 712100, China; College of Enology, College of Food Science and Engineering, Viti-viniculture Engineering Technology Center of State Forestry and Grassland Administration, Shaanxi Engineering Research Center for Viti-Viniculture, Heyang Viti-Viniculture Station, Northwest A&F University, Yangling, Shaanxi 712100, China; State Key Laboratory of Crop Stress Biology in Arid Areas, College of Horticulture, Northwest A&F University, Yangling, Shaanxi 712100, China; Key Laboratory of Horticultural Plant Biology and Germplasm Innovation in Northwest China, Ministry of Agriculture, Northwest A&F University, Yangling, Shaanxi 712100, China; State Key Laboratory of Crop Stress Biology in Arid Areas, College of Horticulture, Northwest A&F University, Yangling, Shaanxi 712100, China; Key Laboratory of Horticultural Plant Biology and Germplasm Innovation in Northwest China, Ministry of Agriculture, Northwest A&F University, Yangling, Shaanxi 712100, China; College of Enology, College of Food Science and Engineering, Viti-viniculture Engineering Technology Center of State Forestry and Grassland Administration, Shaanxi Engineering Research Center for Viti-Viniculture, Heyang Viti-Viniculture Station, Northwest A&F University, Yangling, Shaanxi 712100, China; State Key Laboratory of Crop Stress Biology in Arid Areas, College of Horticulture, Northwest A&F University, Yangling, Shaanxi 712100, China; Key Laboratory of Horticultural Plant Biology and Germplasm Innovation in Northwest China, Ministry of Agriculture, Northwest A&F University, Yangling, Shaanxi 712100, China; College of Enology, College of Food Science and Engineering, Viti-viniculture Engineering Technology Center of State Forestry and Grassland Administration, Shaanxi Engineering Research Center for Viti-Viniculture, Heyang Viti-Viniculture Station, Northwest A&F University, Yangling, Shaanxi 712100, China

## Abstract

Anthocyanins are plant secondary metabolites that have a variety of biological functions, including pigmentation. The accumulation of anthocyanins is regulated by both transcriptional activators and repressors. Studies have shown that bZIP family members act primarily as positive regulators of anthocyanin biosynthesis, but there are few reports of negative regulation. Here, we report that a grapevine (*Vitis vinifera*) bZIP gene from group K, *VvbZIP36*, acts as a negative regulator of anthocyanin biosynthesis. Knocking out one allele of *VvbZIP36* in grapevine with CRISPR/Cas9 promoted anthocyanin accumulation. Correlation analysis of transcriptome and metabolome data showed that a range of anthocyanin biosynthesis genes were activated in *VvbZIP36* mutant plants relative to the wild type, resulting in the accumulation of related metabolites, including naringenin chalcone, naringenin, dihydroflavonols, and cyanidin-3-*O*-glucoside. The synthesis of stilbenes (α-viniferin), lignans, and some flavonols (including quercetin-3-*O*-rhamnoside, kaempferol-3-*O*-rhamnoside, and kaempferol-7-*O*-rhamnoside) was significantly inhibited, and several genes linked to their metabolism were downregulated in the mutant plants. In summary, our results demonstrate that *VvbZIP36* is a negative regulator of anthocyanin biosynthesis that plays a role in balancing the synthesis of stilbenes (α-viniferin), lignans, flavonols, and anthocyanins.

## Introduction

Flavonoids are a family of specialized plant metabolites that includes flavonols, flavones, proanthocyanidins (PAs; also called condensed tannins), and anthocyanins [1]. They accumulate in different organs and tissues of a wide range of plants and have a variety of biological functions [1]. For example, flavonols and PAs provide protection from UV radiation and fungal invasion, respectively [2], and anthocyanins contribute pigmentation to flowers and fruits, thereby attracting insects and animals and promoting pollen and seed dispersal [3]. Flavonoids have also been associated with enhanced resistance to abiotic and biotic stresses [4]. There is increasing evidence that dietary anthocyanins act as powerful antioxidants with beneficial effects on human health, such as the prevention of cardiovascular disease, obesity, diabetes, and cancer [5].

Flavonols, Pas, and anthocyanins are all end-products of the flavonoid pathway, which has been well characterized in many species, such as *Arabidopsis thaliana* [6], tomato (*Solanum lycopersicum*) [7], strawberry (*Fragaria* × *ananassa*) [8], peach (*Prunus persica*) [9], pear (*Pyrus communis*) [10], and grapevine [11]. The flavonoid biosynthetic pathways in these species share common steps, including chalcone synthase (CHS), chalcone isomerase (CHI), flavanone 3-hydroxylase (F3H), and flavonoid 3′-hydroxylase (F3′H) [2]. Subsequently, the formation of flavonols and leucoanthocyanidins from dihydroflavonols is catalyzed by flavonol synthase (FLS) and dihydroflavonol 4-reductase (DFR), respectively [12]. Anthocyanidin synthase (ANS) then converts leucoanthocyanidins to anthocyanidins [12], which are used as substrates by UDP-glucose flavonoid 3-*O*-glucosyltransferase (UFGT), glutathione *S*-transferase (GST), and anthocyanin 3-*O*-glucoside-6′′-*O*-acyltransferase (3AT) to synthesize anthocyanins [11]. PAs are polymers made from flavan-3-ol units, catechin, and epicatechin, which are synthesized from leucoanthocyanidins by leucoanthocyanidin reductase (LAR) and anthocyanidin reductase (ANR) [13].

The biosynthesis of flavonoids is controlled by a complex network of structural and regulatory genes. Many transcriptional activators involved in regulating flavonoid biosynthesis, especially anthocyanins and PAs, have been identified in various species. These include *AtMYB113* and *AtMYB114* (*A. thaliana*) [14], *MdBBX22* and *MdHY5* (bZIP family genes from apple; *Malus domestica*) [15], *FvbHLH9* (strawberry) [16], *PpMYB10* and *PpbHLH64* (pear) [17], *PpNAC1* and *PpMYB10.1* (peach) [18], and *VvMYBA1*, *VvMYBA2*, and *VvMYBPA1* (grapevine) [11, 19]. It has also been determined in multiple species that a transcriptional activation complex of R2R3-MYB, bHLH, and WD-repeat (WD40) proteins regulates the expression of flavonoid biosynthetic genes [4]. In addition to transcriptional activators, transcriptional repressors have been shown to play important roles in regulating flavonoid biosynthesis pathways. For example, heterologous overexpression of strawberry *FaMYB1* in *Lotus corniculatus* inhibits the expression of *LAR* and *ANR* and significantly reduces the content of PAs in leaves [20]; the poplar (*Populus*) MYB182 protein significantly reduces the content of anthocyanins and PAs in poplar by reducing the expression of *DFR1*, *ANS1*, and *ANR1* [21]; and *PpMYB18* acts as a negative regulator, balancing the accumulation of anthocyanins and PAs during peach fruit ripening and at juvenile stages of development [13].

Grapevines (*Vitis vinifera*) are economically important fruit-producing plants rich in flavonoids [22]. The regulation of flavonoid biosynthesis by transcription factors (TFs) has been studied in grapevine, including analyses of *VvMYBPA1*/*VvMYBPA2*, *VvMYBA1*/*VvMYBA2*, *VviMYB86*, *VvBBX44*, and *VvWRKY26* [1,11,19,23–25]. For example, *VvMYBPA1* or *VvMYBPA2* activate the expression of *VvANR* and *VvLAR* and promote PA accumulation [1, 19]. Overexpression of *VvMYBA1* activates the transcription of *Vv3AT* in the white grape variety Chardonnay, causing the fruit to accumulate anthocyanins and turn red, whereas silencing of *VvMYBA1* or *VvMYBA2* in the red grape variety Shiraz results in a significant decrease in fruit anthocyanin content [11]. Notably, bZIP family genes, especially *HY5*, have also been shown to play key roles in the regulation of flavonoid biosynthesis in many species, including *Arabidopsis* [26], apple [27], tomato [28], pear [29], and strawberry [16]. However, there are only a few related studies of bZIP TFs in grapevine [30, 31]. Characterization of the regulation of grapevine flavonoid biosynthesis has mostly centered on gain-of-function experiments, and there are few loss-of-function studies. Thus, much remains to be learned about the transcriptional regulation of flavonoids by bZIP TFs in grapevine, and in this regard, the analysis of new functional knockouts of genes of interest represents a promising experimental strategy.

Genome editing technology has proven effective for loss-of-function research in many horticulturally important crops, such as tomato, tobacco, rice (*Oryza sativa*), wheat (*Triticum aestivum*), and maize (*Zea mays*) [32]. There have been far fewer such studies of fruit tree crops, with the limited examples being grapevine [33], citrus [34], and apple [35]; this is mainly due to their longer genetic transformation cycle and lower transformation efficiency. In citrus, targeted editing of the disease susceptibility gene *CsLOB1* increased resistance to citrus canker [36], and targeting the promoter region of this gene also produced disease-resistant plants [34]. In apple, *clustered regularly interspaced short palindromic repeats* (CRISPR)/Cas9 ribonucleoproteins (RNPs) were successfully delivered to apple protoplasts to edit *DIPM-1*, *DIPM-2*, and *DIPM-4*, thereby increasing fire blight resistance [35]. In grapevine, *VvWRKY52*, *VvMLO3*, and *VvPR4b* were successfully mutated using CRISPR/Cas9, increasing resistance to *Botrytis cinerea*, downy mildew, and powdery mildew, respectively [37–39].

In the current study, we used CRISPR/Cas9 to target the grapevine bZIP family gene *VvbZIP36* and found that it contributes to the regulation of anthocyanin levels by balancing the synthesis of stilbenes (α-viniferin), lignans, flavonols, and anthocyanins.

## Results

### Bioinformatic analysis of *VvbZIP36*

**Figure 1:**
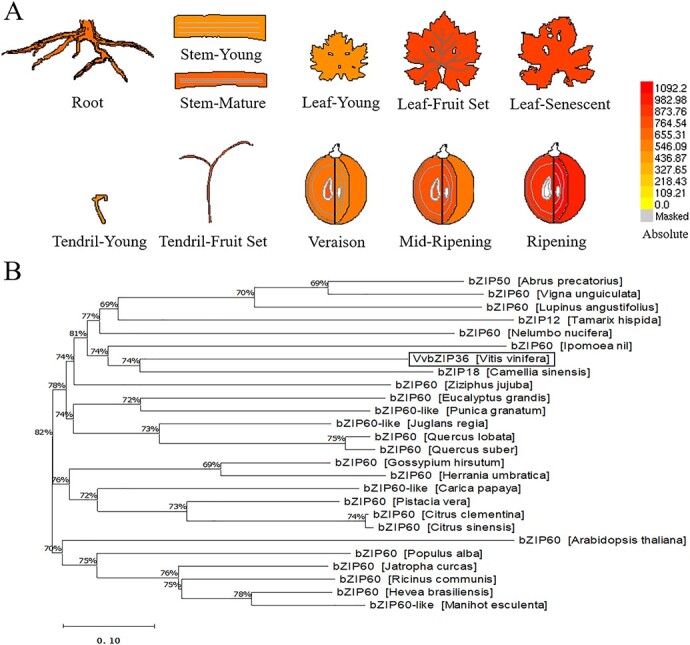
Phylogenetic analysis of VvbZIP36 and expression analysis of *VvbZIP36*. **A** Tissue-specific expression analysis of *VvbZIP36* based on the Grape eFP Browser (http://bar.utoronto.ca/efp_grape/cgi-bin/efpWeb.cgi). The color intensity represents the level of expression. **B** Phylogenetic tree showing the relationship between VvbZIP36 and homologs from other species. All the protein sequences (*Abrus precatorius* bZIP50 [XP_027366621.1], *Arabidopsis thaliana* bZIP60 [OAP18538.1], *Camellia sinensis* bZIP18 [ALL97705.1], *Carica papaya* bZIP60-like [XP_021909218.1], *Citrus clementina* bZIP60 [XP_006435475.1], *Citrus sinensis* bZIP60 [XP_006473869.1], *Eucalyptus grandis* bZIP60 [XP_010063178.2], *Gossypium hirsutum* bZIP60 [XP_016748430.1], *Herrania umbratica* bZIP60 [XP_021283978.1], *Hevea brasiliensis* bZIP60 [XP_021646411.1], *Ipomoea nil* bZIP60 [XP_019172061.1], *Jatropha curcas* bZIP60 [NP_001292941.1], *Juglans regia* bZIP60-like [XP_018832980.1], *Lupinus angustifolius* bZIP60 [XP_019461939.1], *Manihot esculenta* bZIP60-like [XP_021600128.1], *Nelumbo nucifera* bZIP60 [XP_010270593.1], *Pistacia vera* bZIP60 [XP_031270269.1], *Populus alba* bZIP60 [XP_034931370.1], *Punica granatum* bZIP60-like [XP_031377447.1], *Quercus lobata* bZIP60 [XP_030951078.1], *Quercus suber* bZIP60 [XP_023918417.1], *Ricinus communis* bZIP60 [XP_002510740.1], *Tamarix hispida* bZIP12 [AFO63291.1], *Vigna unguiculata* bZIP60 [XP_027938181.1], and *Ziziphus jujuba* bZIP60 [XP_015880016.1]) were downloaded from the National Center for Biotechnology Information (NCBI) (http://blast.ncbi.nlm.nih.gov/Blast.cgi).

In previous studies, 47 bZIP TF-encoding genes were identified from grapevine (*V. vinifera*) [40]. Tissue-specific expression analysis showed that the bZIP gene *VvbZIP36* has high expression in leaves, stems, flowers, tendrils, and especially fruits [40]. In this study, we found that *VvbZIP36* is expressed in the roots, tendrils, stems, leaves, and fruit of grapevine, with the highest expression in mature stems, leaves at fruit set, and senescent leaves, as well as ripening fruit (Fig. 1A), similar to a previous study. According to a phylogenetic analysis using bZIP TF sequences from *Arabidopsis* and rice, we found that *VvbZIP36* was classified in the K subgroup as a homolog of *AtbZIP60* and *OsbZIP74*, which encode proteins with both a bZIP domain and a trans-membrane domain (TMD) [40]. Previous studies showed that both *AtbZIP60* and *OsbZIP74* play important roles in the endoplasmic reticulum (ER) stress response in *Arabidopsis* and rice, respectively [41, 42]. To further analyze the group K bZIP TFs, we searched the National Center for Biotechnology Information (NCBI) (http://blast.ncbi.nlm.nih.gov/Blast.cgi) database for VvbZIP36 homologs and found 25 sequences from other species, all of which contained the bZIP domain and a putative TMD (Fig. S1). When analyzing their phylogenetic relationships, as shown in Fig. 1B, we found that VvbZIP36 was most closely related to *Camellia sinensis* bZIP18 and *Ipomoea nil* bZIP60.

### Knocking out one allele of *VvbZIP36* promotes the accumulation of anthocyanins in grapevine

**Figure 2:**
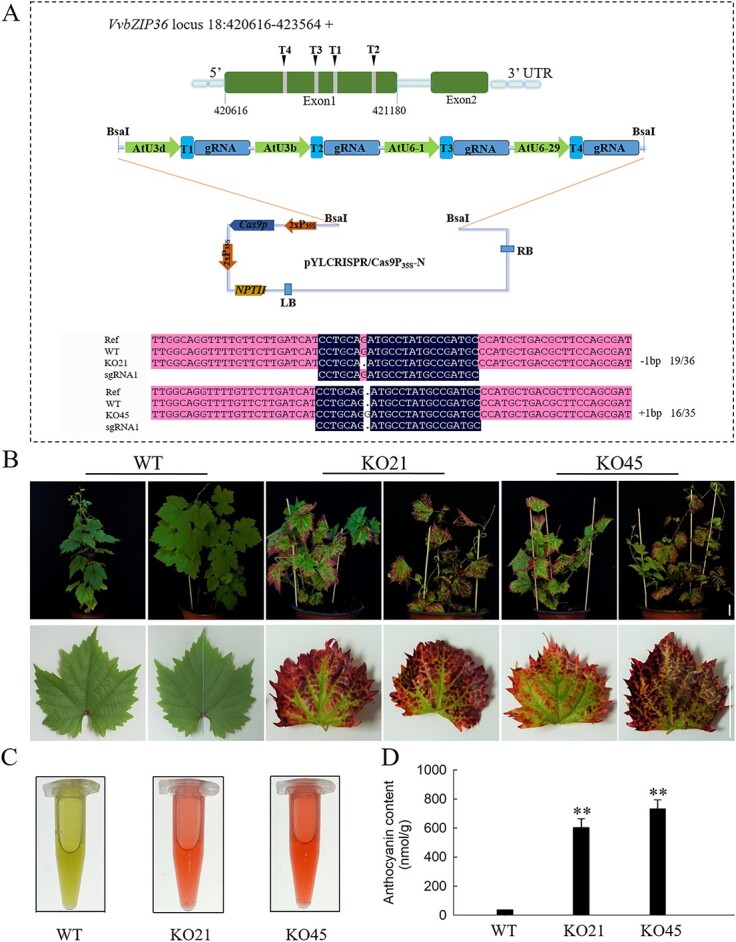
Knocking out one allele of *VvbZIP36* promotes anthocyanin accumulation in grapevine. **A** Targeted mutagenesis of *VvbZIP36* in transgenic lines. “+” represents insertions, and “-” represents deletions. The numbers before and after the “/” represent the number of mutant clones and the total number of clones sequenced. **B** Red leaf phenotype of seven-month-old transgenic (KO21 and KO45) and WT plants. Scale bar = 3 cm. **C** Anthocyanin solutions extracted from 0.3 g of KO21, KO45, and WT leaf tissue. **D** Anthocyanin content in KO21, KO45, and WT leaves. Values are means ± SE (n = 3). Asterisks indicate statistical significance (**P < 0.01, Student’s *t*-test) between the transgenic and WT plants.

In our previous study, we successfully knocked out one allele of *VvbZIP36* in grapevine (Thompson Seedless) using the CRISPR/Cas9 genome editing system and obtained one mutant grapevine plant (KO45) [43]. No off-target events were identified in KO45, as determined by whole-genome sequencing (WGS) [43]. Subsequently, through continuous identification of regenerated lines, 7 transgenic positive plants were identified from 22 regenerated plants (Fig. S2). Among them, the mutant line KO21 was found by Sanger sequencing. As shown in Fig. 2A, we designed four CRISPR/Cas9 sgRNAs for the first exon of *VvbZIP36* [43], but only target 1 was mutated in KO21 and KO45. In addition, the KO21 and KO45 lines were found to contain monoallelic mutations, with a base insertion in KO21 and a base deletion in KO45.

We observed that the leaves of KO21 and KO45 turned red (Fig. 2B) and hypothesized that *VvbZIP36* plays a role in regulating anthocyanin biosynthesis. We therefore collected leaves from the mutant lines (KO21 and KO45) and the wild type (WT) to determine the total anthocyanin contents. As shown in Fig. 2C, D, the anthocyanin levels in the leaves of both KO21 and KO45 were significantly higher than those in the WT.

### Anthocyanin biosynthetic genes are differentially expressed between KO45 and WT plants

**Figure 3:**
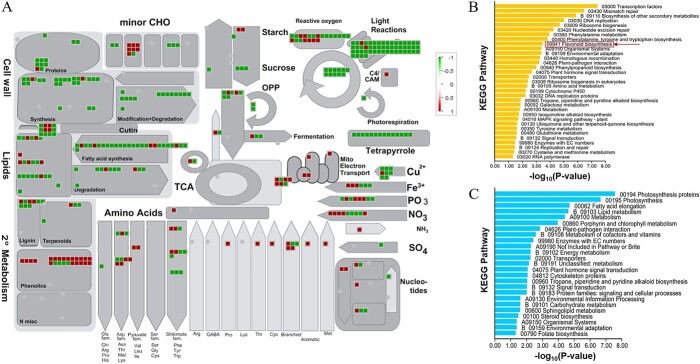
MapMan and KEGG pathway enrichment analysis of differentially expressed genes (DEGs) in the *VvbZIP36* mutant line (KO45). **A** MapMan enrichment analysis of DEGs in the leaves of KO45 compared with the WT. The colored boxes indicate the log2 of the DEG expression ratios. **B**, **C** KEGG pathway enrichment analysis of genes that are expressed at higher levels (**B**) or lower levels (**C**) in leaves of KO45 compared with the WT. The red box represents the flavonoid biosynthesis pathway.

To further analyze the connection between *VvbZIP36* and anthocyanin biosynthesis, we performed an RNA-seq analysis of KO45 (no off-target events certified by WGS [43]) and WT leaves from plants grown in a growth chamber under normal conditions. The results of normalized cluster analysis and Pearson’s correlation analysis show that the biological replicates of each group were highly correlated (Pearson coefficient > 0.9; Fig. S3). A total of 4103 DEGs were identified between KO45 and the WT, including 1965 upregulated and 2138 downregulated genes (Fig. S4). As shown in Fig. 3A, the gene ontology MapMan analysis revealed that most of the downregulated DEGs were related to “photosynthesis”, “tetrapyrrole”, and “cell wall”. By contrast, DEGs involved in “lipid synthesis”, “fatty acid synthesis”, “amino acid metabolism”, and “reactive oxygen” were both upregulated and downregulated. Finally, most of the DEGs related to phenolic compound metabolism were upregulated. This information is summarized in Supplementary Data S1.

Subsequently, we performed KEGG pathway enrichment analysis of the 4103 DEGs and found that “transcription factors” and the “mismatch repair”, “secondary metabolite biosynthesis”, “DNA replication”, “phenylalanine metabolism”, “flavonoid biosynthesis”, and “phenylpropanoid biosynthesis” pathways were significantly enriched in the 1965 upregulated genes (Fig. 3B). By contrast, the “photosynthesis”, “fatty acid elongation”, “lipid”, “vitamin and energy metabolism”, “transporters”, and “signal transduction” pathways were significantly enriched in the 2138 downregulated genes (Fig. 3C). Through further analysis, we found that most of the genes related to phenolic compounds were associated with the flavonoid biosynthesis pathway (Fig. S5). We identified 28 genes (including chalcone synthase, *CHS*; chalcone isomerase, *CHI*; flavanone 3-hydroxylase, *F3H*; flavonoid 3′-hydroxylase, *F3′H*; dihydroflavonol 4-reductase, *DFR*; flavonol synthase, *FLS*; and anthocyanidin synthase, *ANS*) related to flavonoid biosynthesis based on the KEGG and MapMan enrichment analysis results (Table 1).

**Table 1:** List of differentially expressed genes (DEGs) (with *P*-value <0.05, fold change >2) between WT and CRISPR/Cas9-mediated *VvbZIP36* mutant plants (KO45) related to the flavonoid biosynthesis pathway identified from the transcriptome data

**Gene ID**	**Symbol**	**Description**	***P*-value**	**Log**_**2**_ **FC** (KO45 / WT)
VIT_06s0004g02620	VvPAL1	phenylalanine ammonia-lyase 1	2.30E−82	1.62
VIT_16s0039g01100	VvPAL2	phenylalanine ammonia-lyase 2	2.20E−08	2.26
VIT_11s0016g01640	VvPAL3	phenylalanine ammonia-lyase 3	0.001	1.83
VIT_11s0016g01660	VvPAL4	phenylalanine ammonia-lyase 4	5.49E−07	2.90
VIT_11s0016g01520	VvPAL5	phenylalanine ammonia-lyase 5	1.00E−04	1.38
VIT_18s0001g12800	VvDFR	Dihydroflavonol 4-reductase	1.50E−141	2.29
VIT_16s0098g00860	—	Type-I flavone synthase	1.67E−59	2.6
VIT_13s0047g00210	—	Type-I flavone synthase	1.50E−39	1.69
VIT_04s0023g03370	VvF3H1	Flavanone 3-hydroxylase 1	1.06E−86	2.09
VIT_18s0001g14310	VvF3H2	Flavanone 3-hydroxylase 2	1.80E−99	2.13
VIT_17s0000g07210	VvF3’H1	Flavonoid 3′ hydroxylase 1	1.63E−09	1.25
VIT_17s0000g07200	VvF3’H3	Flavonoid 3′ hydroxylase 3	9.22E−41	1.11
VIT_08s0007g05160	VvF3’5’H2	Flavonoid 3′,5′-hydroxylase 2	3.52E−13	2.2
VIT_13s0067g03820	VvCHI1	Chalcone isomerase 1	3.20E−191	2.52
VIT_13s0067g02870	VvCHI2	Chalcone isomerase 2	3.40E−105	2.27
VIT_14s0068g00920	VvCHS1	Chalcone synthase 1	1.40E−128	2.23
VIT_14s0068g00930	VvCHS2	Chalcone synthase 2	8.48E−78	1.7
VIT_05s0136g00260	VvCHS3	Chalcone synthase 3	4.60E−203	2.95
VIT_02s0025g04720	VvANS	Anthocyanidin synthase	5.15E−97	1.94
VIT_01s0011g02960	VvLAR1	Leucoanthocyanidin reductase 1	1.97E−72	1.93
VIT_00s0361g00040	VvANR	Anthocyanidin reductase	1.70E−145	2.37
VIT_09s0002g08090	VvFLS1	Flavonol synthase 1	7.06E−40	1.8
VIT_18s0001g03510	VvFLS2	Flavonol synthase 2	0.007	−1.78
VIT_08s0105g00380	VvFLS3	Flavonol synthase 3	0.009	1.62
VIT_03s0091g01080	VvFLS4	Flavonol synthase 4	2.17E−06	−1.4
VIT_18s0001g03430	VvFLS5	Flavonol synthase 5	2.41E−21	1.44
VIT_06s0009g02010	VvFLR	Flavonol-3-*O*-rhamnosyltransferase	3.17E−09	−7.71
VIT_06s0009g01990	VvFLR	Flavonol-3-*O*-rhamnosyltransferase	3.63E−13	−8.97

### Metabolome profiling indicates that *VvbZIP36* regulates anthocyanin biosynthesis in grapevine

To further investigate the function of *VvbZIP36*, we compared the metabolomes of the leaves of transgenic (KO45) and WT plants. The results of normalized cluster analysis and Pearson’s correlation analysis show that the biological replicates of each group are highly correlated (Pearson coefficient > 0.9; Fig. S6). A total of 347 differentially abundant metabolites between KO45 and the WT were identified and quantified; 185 were more abundant in KO45 than in the WT, and 162 were less abundant (Fig. S7). Of the top 20 metabolites showing higher levels in KO45, most were flavones, triterpene, flavanols, anthocyanidins, organic acids, and stilbenes, whereas the 20 that showed the greatest reduction were mainly flavonols, lignans, organic acids, amino acids and derivatives, and phenolic acids (Table 2).

**Table 2:** List of the top 20 differentially abundant metabolites in the leaves of CRISPR/Cas9-mediated *VvbZIP36* mutant (KO45) and WT plants in a widely targeted metabolome data set

**Compound**	**Class**	***P*-value**	**Log**_**2**_ **FC** (KO45 / WT)
Luteolin-7-*O*-glucoside (Cynaroside)*	Flavones	0.004	16.58
Corosolic acid	Triterpenes	0.003	15.79
Maslinic acid	Triterpenes	0.003	15.76
3,24-Dihydroxy-17,21-semiacetal-12(13)oleanolic fruit	Triterpenes	0.004	15.59
2-Hydroxyoleanolic acid	Triterpenes	0.004	15.26
Alphitolic acid	Triterpenes	0.016	15.26
6-Aminocaproic acid	Organic acids	0.049	14.36
3′-*O*-Methyl-(−)-epicatechin	Flavanols	0.015	14.33
Galangin-7-*O*-glucoside	Flavones	0.027	12.69
Cyanidin-3-*O*-(6′′-*O*-acetyl)glucoside	Anthocyanidins	0.012	12.63
Luteolin-7-*O*-rutinoside	Flavones	0.094	11.88
Poncirin (Isosakuranetin-7-*O*-neohesperidoside)	Flavanones	0.007	11.39
trans-Trimethoxyresveratrol	Stilbenes	0.008	11.29
Resveratrol-3,5-di-*O*-glucoside	Stilbenes	0.014	10.39
3,11-Dioxo-19α-hydroxyurs-12-en-28-oic acid	Triterpenes	0.003	9.57
12,13-Dihydroursolic acid	Triterpenes	0.001	7.94
Pomolic acid	Triterpenes	0.001	5.87
5-O-Feruloylquinic acid	Phenolic acids	0.027	4.93
4-Guanidinobutyric acid	Organic acids	0.001	4.82
Apigenin-7-*O*-neohesperidoside (Rhoifolin)	Flavones	0.001	4.30
Quercetin-3-*O*-rhamnoside (Quercitrin)	Flavonols	0.002	−16.06
Kaempferol-3-*O*-rhamnoside (Afzelin) (Kaempferin)*	Flavonols	0.013	−13.32
Kaempferol-7-*O*-rhamnoside*	Flavonols	0.014	−12.57
Isolariciresinol-9-*O*-glucoside	Lignans	0.003	−12.32
3-Guanidinopropionic acid	Organic acids	0.011	−11.91
Feruloylferuloyltartaric acid	Phenolic acids	0.016	−11.35
γ-Glutamylmethionine	Amino acids and derivatives	0.008	−10.76
4′-Methoxycatalposide	Monoterpenoids	0.006	−9.82
“Eriodictyol-7-*O*-(6″“-*O*-*p*-coumaroyl)glucoside”	Flavanones	0.023	−8.7
α-Viniferin	Stilbenes	0.043	−8.54
Taxifolin-3-*O*-rhamnoside (Astilbin)	Flavanonols	0.006	−7.47
3-Hydroxyphloretin-4’-*O*-(4″,6″-di-*O*-galloyl)glucoside	Chalcones	0.022	−6.82
Pyridoxine	Vitamins	0.001	−5.20
2,4,6-Tri-*O*-galloyl-D-glucose	Phenolic acids	0.082	−4.33
Pyridoxine-5′-*O*-glucoside	Vitamins	0.013	−4.20
Engeletin	Flavanonols	0.001	−3.89
2′-Deoxyadenosine-5′-monophosphate	Nucleotides and derivatives	0.001	−3.75
Jasmonic acid	Organic acids	0.001	−3.70
Methyl *L*-pyroglutamate	Pyrrole alkaloids	0.005	−3.49
4,7,9,9′-Tetrahydroxy-3,3′-dimethoxy-8-O-4′-neolignan	Others	0.006	−3.29

**Figure 4:**
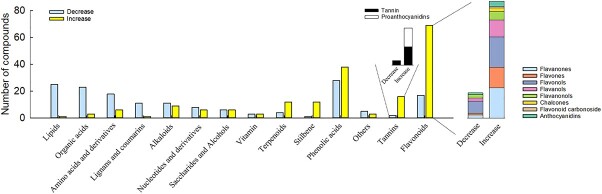
Statistical analysis of differentially abundant metabolites in the leaves of KO45 compared with the WT. Blue represents metabolites that are present at lower levels in KO45 compared with the WT, and yellow represents metabolites that are present at higher levels in KO45.

**Figure 5:**
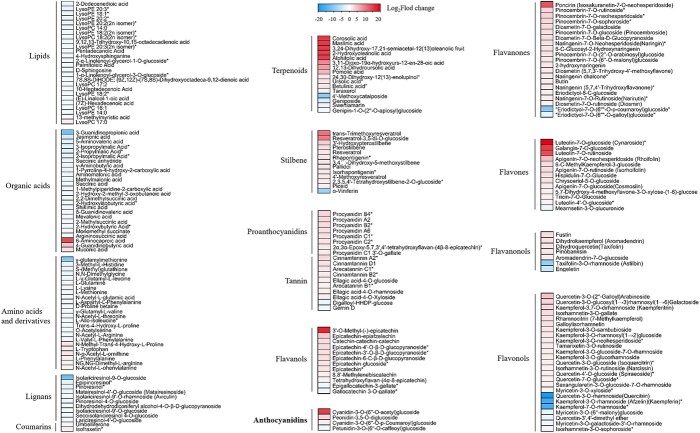
Heat map analysis of differentially abundant metabolites. Red represents more abundant metabolites, and green represents less abundant metabolites in the mutant line compared with the WT; the color intensity reflects the degree of metabolite accumulation.

KEGG enrichment analysis of the differentially abundant metabolites revealed terms that were mainly clustered around amino acids and derivatives, organic acids, lignans, lipids, stilbene, terpenoids, tannin, flavonoids, and phenolic acid pathways (Fig. S8). We noted that metabolites associated with lipids, organic acids, lignans, and amino acids and derivatives showed reduced levels in KO45 leaves, whereas metabolites related to terpenoid, stilbene, tannin, and flavonoid pathways had increased levels (Fig. 4, 5). As shown in Fig. 5, the content of all the PAs from the tannin category increased in KO45 relative to the WT, and the flavonoids that increased in KO45 included mainly flavanones, flavones, flavanonols, flavanols, anthocyanidins, and flavonols. Among the four differentially abundant anthocyanidins, levels of cyanidin-3-*O*-(6′′-*O*-acetyl)glucoside, cyanidin-3-*O*-(6′′-*O*-p-coumaroyl)glucoside, and peonidin-3,5-*O*-diglucoside were higher in KO45 than in the WT (Table 2), whereas the content of petunidin-3-*O*-(6′′-*O*-caffeoyl) glucoside was lower in KO45 (Fig. 5). In the flavonol category, quercetin-3-*O*-rhamnoside, kaempferol-3-*O*-rhamnoside, and kaempferol-7-*O*-rhamnoside levels were significantly lower in KO45, and they showed the greatest reduction among the 162 decreased differentially abundant metabolites (Fig. 5 and Table 2).

### *VvbZIP36* is important in anthocyanin biosynthesis regulation

**Figure 6:**
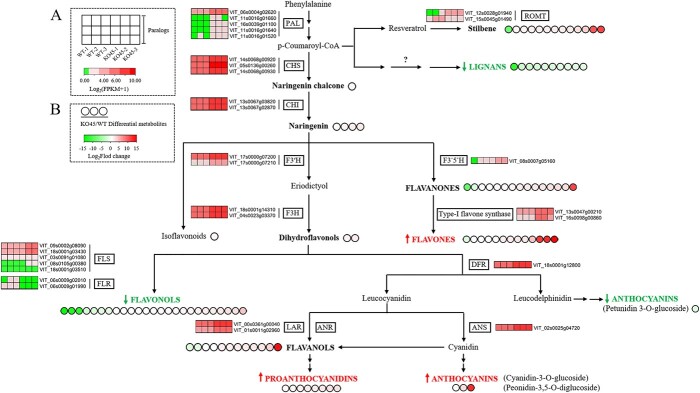
The mechanism of *VvbZIP36* involvement in anthocyanin biosynthesis. **A** The square heat map represents the transcriptional regulation of differentially expressed genes (DEGs) related to phenylpropanoid metabolism in transgenic (KO45) and WT plants. Normalized gene expression values [log_2_(FPKM+1)] were used to plot the heat maps. **B** The circular heat maps represent the relative accumulation levels of differentially abundant metabolites related to phenylpropanoid metabolism in KO45 and WT plants. The log_2_FC (fold change) values were used to plot the heat maps.

To further understand the *VvbZIP36*-associated regulatory network involved in modulating anthocyanin biosynthesis, we performed a correlation analysis of the transcriptome and metabolome data. The expression of genes in the anthocyanin biosynthesis pathway (five *PAL*, three *CHS*, two *CHI*, two *F3′H*, two *F3H*, one *DFR*, and one *ANS* gene) was generally higher in KO45 than in WT, and the corresponding metabolites, such as naringenin chalcone, naringenin, dihydroflavonols, and anthocyanins, were more abundant to varying degrees (Fig. 6).

In other branches of the flavonoid biosynthesis pathway, such as flavanones and flavones, the levels of most metabolites were significantly higher in KO45 than in the WT. Correspondingly, the expression of *VvF3′5′H2* (flavonoid 3′,5′-hydroxylase 2: VIT_08s0007g05160) and two Type-I flavone synthase genes (VIT_16s0098g00860, VIT_13s0047g00210) was higher. The PAs and most of their precursor metabolites (flavanols) also accumulated to higher levels in KO45 than in the WT, accompanied by higher expression levels of *VvLAR1* (leucoanthocyanidin reductase 1: VIT_01s0011g02960) and *VvANR* (anthocyanidin reductase: VIT_00s0361g00040). The content of some flavonols was higher in KO45 than in the WT, whereas the abundance of others was lower (Table 2 and Fig. 6), accompanied by higher expression of three *FLS* genes (*VvFLS1*: VIT_09s0002g08090; *VvFLS3*: VIT_08s0105g00380; *VvFLS5*: VIT_18s0001g03430) and lower expression of two *FLS* genes (*VvFLS2*: VIT_18s0001g03510; *VvFLS4*: VIT_03s0091g01080) and two *FLR* genes (flavonol-3-*O*-rhamnosyltransferase: VIT_06s0009g02010, VIT_06s0009g01990).

Interestingly, all the differentially abundant lignan metabolites were lower in KO45 than in the WT, whereas five *PAL* genes from the early general phenylpropanoid pathway were expressed at higher levels in KO45 (Fig. 6). Notably, we did not identify DEGs involved in lignan biosynthesis in the KEGG pathway enrichment analysis. In the stilbene biosynthesis pathway, two *ROMT* genes (trans-resveratrol di-*O*-methyltransferase: VIT_12s0028g01940, VIT_15s0045g01490) were identified, one of which showed higher expression in KO45 than in the WT, whereas the other showed lower expression.

**Figure 7:**
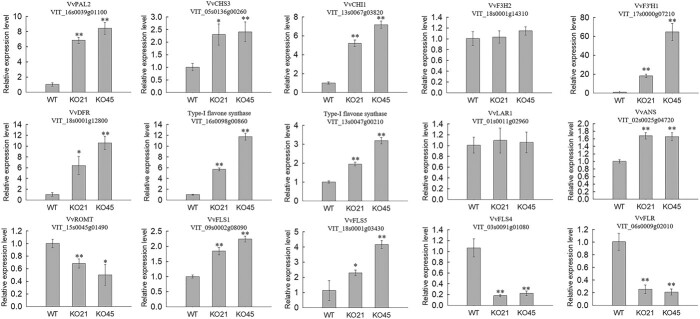
Quantitative real time (qRT)-PCR analysis of differentially expressed genes (DEGs) related to phenylpropanoid metabolism in the leaves of transgenic lines (KO21 and KO45) and WT plants. Values are means ± SE (n = 3). Asterisks indicate statistical significance (^**^P < 0.01, ^*^0.01 < P < 0.05, Student’s *t*-test) between the transgenic and WT plants.

Subsequently, we randomly selected 15 of the 30 DEGs involved in the phenylpropanoid pathway from Fig. 6 for confirmatory qRT-PCR expression analysis. As shown in Fig. 7, with the exceptions of *VvF3H2* and *VvLAR1*, their expression patterns were consistent with the transcriptome data.

## Discussion

The basic region/leucine zipper (bZIP) TFs are important regulators in plants and are involved in regulating many metabolic processes, such as energy metabolism, development, senescence, light signaling, anthocyanin biosynthesis, and response to biotic and abiotic stimuli [26, 44]. In previous studies, 47 bZIP TFs were identified from grapevine [40]. Tissue specific-expression analysis showed that *VvbZIP36* was highly expressed in leaves, stems, flowers, tendrils, and especially fruits [40]. Similar results were found in this study (Fig. 1A). Because previous reports indicate that bZIP TFs play an important role in the synthesis of anthocyanin [26–31], we speculated that *VvbZIP36* might be involved in the regulation of anthocyanin biosynthesis. Knocking out one allele of *VvbZIP36* did not affect expression (Fig. 2A, Fig. S9) but caused 46.45% of the transcripts of this gene to be mutated (Table S1). This resulted in a significant accumulation of anthocyanins and a red leaf phenotype in white grapevine (Thompson Seedless) (Fig. 2B-D), suggesting that *VvbZIP36* is a negative regulator. Previously, most bZIP genes reported to be involved in regulating anthocyanin biosynthesis have been described as positive regulators, including *MdbZIP44* and *MdHY5* in apple [27, 45], *SlHY5* in tomato [7, 28], and *PybZIPa* and *PyHY5* in pear [10, 29]. Similar results were previously reported from studies of grapevine: *VvibZIPC22* was observed to promote the accumulation of several flavonoids by regulating the expression of *CHS* and *FLS* [30], and *VvHY5* promotes flavonol biosynthesis by activating the expression of an *FLS* [31]. These results indicate that *VvbZIP36* may have a different regulatory framework from other bZIP TFs that control the anthocyanin biosynthesis pathway. Previously, most studies on K subgroup genes (including *AtbZIP60* and *OsbZIP74*) have been related to endoplasmic reticulum stress [41, 42]. This is the first report to show that K subgroup genes (such as *VvbZIP36*) participate in plant anthocyanin biosynthesis.

To further analyze the regulatory mechanism by which *VvbZIP36* influences anthocyanin biosynthesis, we performed transcriptome analysis of the leaves of a CRISPR/Cas9-mediated *VvbZIP36* mutant line (KO45) and WT plants. A total of 1965 and 2138 genes were expressed at higher and lower levels, respectively, in KO45 compared with the WT (Fig. S4). Genes expressed at lower levels in the transgenic leaves were mainly involved in photosynthesis, cell wall and lipid metabolism, and the fatty acid synthesis pathway (Fig. 3), suggesting that *VvbZIP36* may positively regulate these synthetic pathways, consistent with similar studies. For example, in *Arabidopsis*, *AtHY5* promotes photosynthetic pigment accumulation in response to light [46]. In soybean, *GmbZIP04g* and *GmbZIP07g* can transcriptionally regulate the expression of *GmRCAα*, a key photosynthetic gene, by binding to its promoter [47]. Moreover, overexpression of *NsbZIP1* and *HSbZIP1* significantly promotes the synthesis of lipids in *Nannochloropsis salina* [48] and *Chlorella* sp. HS2 [49], respectively. In addition, research shows that *MabZIP93* can activate the expression of cell wall modifying genes during banana (*Musa*) fruit ripening [50]. Given this association of *VvbZIP36* with photosynthesis and the central role that photosynthesis plays in plant growth and development [51], it is perhaps not surprising that we only obtained transgenic *VvbZIP36* knock-out grapevine lines with single allele mutations (Fig. 2A, Table S1): complete *VvbZIP36* knockout may be lethal.

We identified an association between the DEGs that were expressed at higher levels in KO45 and the flavonoid metabolism pathway (Fig. 3), consistent with the observed phenotype (Fig. 2). Further analysis revealed that this subset included genes in the core anthocyanin biosynthesis pathway, including *PAL*, *CHS*, *CHI*, *F3H*, *F3′H*, *DFR*, *FLS*, and *ANS* (Table 1).

Metabolome analysis showed that a total of 185 metabolites were more abundant in KO45 than in the WT, and 162 were less abundant (Fig. S7). Those that were less abundant belonged to the lipid, organic acid, lignan, and amino acids and derivatives categories, whereas those that were more abundant belonged to the terpenoid, stilbene, tannin, and flavonoid categories (Fig. 4, 5). Interestingly, we found that most of the genes related to terpenoids were downregulated (Fig. 3A), but the corresponding levels of most triterpene metabolites were higher in KO45 than in the WT (Table 2). We speculated that the metabolism and regulatory network of triterpenes in mutant plants may be very complicated, or some terpenoid-related genes may not have been discovered, leading to this result.

Stilbenes, lignans, and flavonoids are all synthesized from the same precursor metabolite, *p*-coumaroyl-CoA [52]. Subsequently, correlation analysis of the transcriptome and metabolome data showed that many genes involved in the anthocyanin biosynthesis pathway, including *PAL*, *CHS*, *CHI*, *F3′H*, *DFR*, and *ANS* were expressed at higher levels in KO45 than in the WT, and many related metabolites (including naringenin chalcone, naringenin, dihydroflavonols, and the anthocyanins cyanidin-3-*O*-(6′′-*O*-acetyl)glucoside, cyanidin-3-*O*-(6′′-*O*-p-coumaroyl)glucoside and peonidin-3,5-*O*-diglucoside) were more abundant in KO45 (Fig. 6). Naringenin chalcone, naringenin, and dihydroflavonol metabolites are all important intermediates in anthocyanin biosynthesis, which is part of the flavonoid pathway [2, 12]. Our data suggest that *VvbZIP36* can suppress the expression of key anthocyanin biosynthesis genes; however, Qiu et al (2019) found that knocking out *SlHY5* (a bZIP gene) in tomato can reduce anthocyanin levels and expression of these anthocyanin biosynthesis genes [28]. Similar studies have also been performed on grapevine with the same results [30, 31]. This suggests that bZIP proteins can operate via different mechanisms in regulating anthocyanin biosynthesis. Interestingly, we found that the abundance of the anthocyanin compound petunidin-3-*O*-(6′′-*O*-caffeoyl)glucoside was lower in KO45 than in the WT (Fig. 5), indicating that *VvbZIP36* may be involved in regulating the balance of different anthocyanin compounds. The identification of genes involved in petunidin-3-*O*-(6′′-*O*-caffeoyl)glucoside synthesis will be an interesting area of future study.

**Figure 8:**
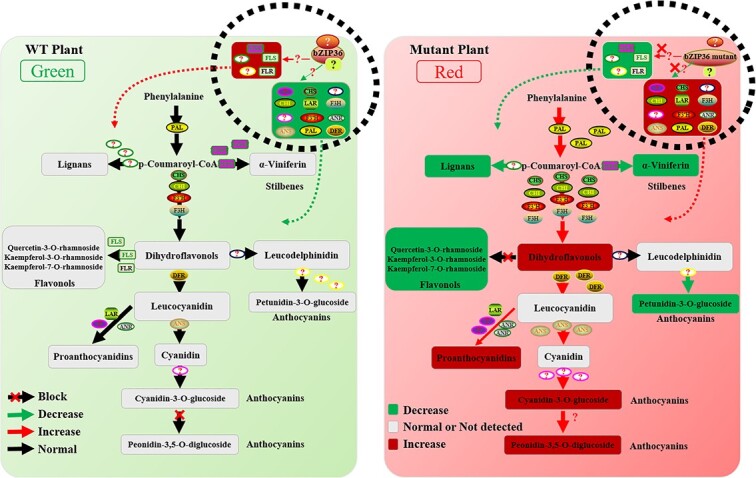
Proposed mechanism by which *VvbZIP36* regulates anthocyanin accumulation in mutant and WT plants. **A** In the WT, there are high levels of expression of biosynthetic genes involved in lignan, α-viniferin, petunidin-3-*O*-glucoside, and flavonol (quercetin-3-*O*-rhamnoside, kaempferol-3-*O*-rhamnoside, and kaempferol-7-*O*-rhamnoside) formation and low expression of genes involved in proanthocyanin (PA) and anthocyanin (cyanidin-3-*O*-glucoside and peonidin-3,5-*O*-diglucoside) biosynthesis. Expression is maintained by *VvbZIP36* through a complex regulatory mechanism, allowing more lignans, α-viniferin, petunidin-3-*O*-glucoside, and flavonols (quercetin-3-*O*-rhamnoside, kaempferol-3-*O*-rhamnoside, and kaempferol-7-*O*-rhamnoside) and fewer PAs and anthocyanins (cyanidin-3-*O*-glucoside and peonidin-3,5-*O*-diglucoside) to be synthesized. This causes leaf anthocyanin content to be maintained at a low level and the leaves to appear green. **B** In *VvbZIP36* mutant plants, the expression of biosynthesis genes involved in lignan, α-viniferin, petunidin-3-*O*-glucoside, and flavonol (quercetin-3-*O*-rhamnoside, kaempferol-3-*O*-rhamnoside, and kaempferol-7-*O*-rhamnoside) biosynthesis was inhibited, whereas anthocyanin synthesis genes were activated. This caused the synthesis of lignans, α-viniferin, petunidin-3-*O*-glucoside, and flavonols (quercetin-3-*O*-rhamnoside, kaempferol-3-*O*-rhamnoside, and kaempferol-7-*O*-rhamnoside) to decrease and the synthesis of PAs and anthocyanins (cyanidin-3-*O*-glucoside and peonidin-3,5-*O*-diglucoside) to increase. The anthocyanin content in the leaves was maintained at a high level, and the leaves appeared red.

We found that when the anthocyanin biosynthetic pathway was induced, the abundance of various flavonoid components (flavonols, flavanols, PAs, flavanones, and flavones) increased significantly in the mutant lines (Fig. 6). However, we also found that some flavonols, flavanols, flavanones, and flavones were present at lower levels in the mutant lines (Fig. 5), especially flavonols (quercetin-3-*O*-rhamnoside, kaempferol-3-*O*-rhamnoside, and kaempferol-7-*O*-rhamnoside) (Table 2). Correspondingly, the expression of four genes from the flavonol synthesis pathway (*VvFLS2*, *VvFLS4*, and two *VvFLR* genes) was lower in the transgenic plants. These results suggest that *VvbZIP36* is involved in balancing the synthesis of flavonols and anthocyanins. Similar results have also been found in the stilbene (α-viniferin) and lignan biosynthetic pathways, which share the same precursor metabolite, *p*-coumaroyl-CoA, as the precursor of flavonoids, naringenin chalcone. We infer from our results that the action of *VvbZIP36* causes more *p*-coumaroyl-CoA to be used to synthesize stilbenes (α-viniferin) and lignans, and more dihydroflavonols to be synthesized into flavonols (quercetin-3-*O*-rhamnoside, kaempferol-3-O-rhamnoside, kaempferol-7-*O*-rhamnoside, etc.), thereby restricting the synthesis of naringenin chalcone and anthocyanins, ultimately leading to relatively low levels of anthocyanin accumulation. By contrast, more anthocyanin accumulation was seen in mutant plants because of the inhibition of flavonol, stilbene (α-viniferin), and lignan synthesis (Fig. 8). That knockout of one allele of *VvbZIP36* caused such a remarkable phenotype is surprising. A reasonable explanation is that *VvbZIP36* plays a complicated role in the phenylpropanoid pathway. It is an inhibitor for anthocyanins but an activator for metabolites such as flavonols and lignans. Single allele mutations lead to weakened inhibition of anthocyanin synthesis and promotion of stilbene, flavonol, and lignan synthesis. Finally, this caused significant accumulation of anthocyanins in leaves. Second, this may be caused by a cumulative effect. Single allele knockout only partially inhibits or activates downstream genes, but these genes will continue to function for a period of time after expression. This is consistent with the phenotype we observed, in which leaf redness gradually deepened with increasing leaf age (Fig. 2B).

In conclusion, the results reported here indicate that *VvbZIP36* contributes to the regulation of anthocyanin biosynthesis by balancing the synthesis of stilbenes (α-viniferin), lignans, flavonols, and anthocyanins in grapevine. This study provides new insights into the regulation of anthocyanin biosynthesis that will be valuable for grapevine breeding programs.

## Materials and methods

### Phylogenetic and expression profile analyses

To investigate the relationship between VvbZIP36 and other bZIP TFs, multiple amino acid sequence alignments were generated using DNAMAN software (Version 5.2.2.0, Lynnon Biosoft, USA) with default parameters, and a phylogenetic analysis was carried out using the neighbor-joining (NJ) method and MEGA software (version 11.0.8), as previously described [53].

The tissue-specific expression of *VvbZIP36* was analyzed using the Grape eFP Browser (http://bar.utoronto.ca/efp_grape/cgi-bin/efpWeb.cgi).

### Plant materials and growth conditions

Vegetative propagation of mutant lines 21 and 45 (KO21 and KO45) and WT grapevine (*V. vinifera* L. cv. Thompson Seedless) was performed as described in a previous study [54]. Two-month-old transgenic and WT plantlets were transferred to plastic pots (10 × 10 × 8 cm) containing a mixture of soil (Pindstrup, Denmark) and vermiculite (1: 1, v: v). After one month of adaptation, the plants were transferred to larger plastic pots (diameter, 15 cm and height, 20 cm) filled with a mixture of soil and vermiculite (1: 1, v: v) and cultivated in a growth incubator (25°C, 16-h photoperiod, and a light intensity of 200 μmol m^−2^ s^−1^; #G2-1000D3, Youke, China) for four months.

### Detection of on-target mutations by Sanger sequencing

Identification of mutant lines was performed as previously described [37, 43]. Specific primers (NPTII-F: 5′-AGA GGC TAT TCG GCT ATG ACT G-3′; NPTII-R: 5′-CAA GCT CTT CAG CAA TAT CAC G-3′) were used to identify stable transgenic lines. Mutant type was identified using *VvbZIP36* gene-specific primers (*VvbZIP36*-Target-F: 5′-ATG GAC GAT TTG GAA ATT GGG G-3′; *VvbZIP36*-Target-R: 5′-TCA CAC CAA AAC TCC ATG AG-3′). The PrimeSTAR Max DNA Polymerase kit (Takara, China) was used to amplify potential mutation regions in the first exon of *VvbZIP36*, and the PCR products were inserted into the pClone007 Simple Vector (TSINGKE, China) to select single clones for sequencing.

### Determination of total anthocyanin contents

The anthocyanin contents of leaves from three seven-month-old transgenic (KO21 and KO45) and WT plants, grown in a growth chamber (#G2-1000D3, Youke, China), were measured after sampling the third to fourth leaf from the top of the plants. The total anthocyanin content was calculated according to Li et al. (2012) [55]. In brief, leaves (approximately 0.3 g) were incubated in 5 mL of 0.1% (v/v) methanol-HCl in the dark at room temperature, and the absorbance of each extract was measured at 530, 620, and 650 nm with a spectrophotometer (UV2600; Unico, China). The experiment was repeated independently three times.

### RNA-seq and data analyses

The third to fourth leaves from the tops of seven-month-old transgenic (KO45) and WT plants were collected, and their transcriptomes were characterized by RNA-seq analysis. Samples from three plants per line were pooled and used as one independent sample, and three independent samples were analyzed per genotype. Total RNA was extracted from the transgenic and WT leaves using TRIzol reagent (Invitrogen, USA). The RNA library preparation and sequencing were performed by Metware Biotechnology Co., Ltd. (Wuhan, China). In brief, RNA-seq libraries were generated using the NEBNext Ultra RNA Library Prep Kit for Illumina (NEB, USA) following the manufacturer’s instructions before sequencing on the Illumina HiSeq platform. The original data were filtered using fastp (v0.19.3) to remove reads with adapter sequences. The resulting cleaned reads were mapped to the *V. vinifera* reference genome (PN40024) (http://plants.ensembl.org/index.html) using HISAT (v2.1.0). FPKM was calculated using featureCounts (v1.6.2), and differential gene expression was determined using DESeq2 (v1.22.1) with a threshold of corrected *P*-value < 0.05 and absolute log_2_FC (fold change) > 1.

All differentially expressed genes (DEGs) were imported into MapMan software (v3.5.1R2; https://mapman.gabipd.org/home) for pathway analysis. The Kyoto Encyclopedia of Genes and Genomes (KEGG) pathway database (https://www.genome.jp/kegg) was used for gene functional annotation, and KEGG enrichment analysis was performed using TBtools software [56]. All normalized cluster analyses and Pearson’s correlation analyses were performed using Metware Cloud (https://cloud.metware.cn/#/tools/tool-list).

### Metabolome analysis

The third to fourth leaves from the tops of three seven-month-old transgenic (KO45) and WT plants were collected for a widely targeted metabolome analysis. Approximately 5 g of leaf tissue per line was used as one independent sample, and three independent samples were analyzed. Sample extraction, metabolite identification, and quantification were performed by Metware Biotechnology Co., Ltd. (Wuhan, China). An ultra-performance liquid chromatography-electrospray tandem mass spectrometry (UPLC*-*ESI*-*MS*/*MS) system (UPLC, SHIMADZU Nexera X2) was used to analyze sample extracts as described previously [57]. Metabolites with a variable importance in projection (VIP) ≥ 1 and an absolute log_2_FC (fold change) ≥ 1 were considered to be differentially regulated between groups.

### Quantitative real time (qRT)-PCR analysis

For gene expression analysis, RNA was extracted from the leaves of seven-month-old transgenic (KO21 and KO45) and WT plants grown in a growth chamber using the E.Z.N.A. Plant RNA Kit (#R6827-01, Omega Bio-tek, USA). First-strand cDNA was synthesized using the HiScript II Q RT SuperMix for qPCR(+g DNA wiper) Kit (#R223-01, Vazyme) according to the manufacturer’s protocol. qRT-PCR was performed using the SYBR qPCR Master Mix (#Q311-02, Vazyme) on a StepOnePlus RT-PCR instrument (Thermo Fisher Scientific), and the gene-specific primers used are listed in Supplementary Table S2.

### Statistical analysis

Data analysis was performed using Excel 2016 software (Microsoft Corporation, USA). Data were plotted using SigmaPlot (v14.0, Systat Inc., CA, USA). Tests for significant differences were performed using SPSS statistics software (v25.0, IBM China Company Ltd., Beijing, China).

## Supplementary Material

Web_Material_uhac022

## Data Availability

The sequence data have been submitted to the NCBI Sequence Read Archive under BioProject ID PRJNA773187.
