# Supplementary material for: CRISPR/Cas9-mediated mutagenesis of VvbZIP36 promotes anthocyanin accumulation in grapevine (Vitis vinifera)
Source: Hortic Res. 2022 Feb 20;9:uhac022. doi: 10.1093/hr/uhac022 (PMC9174745; doi:10.1093/hr/uhac022)
Supplement: Web_Material_uhac022 [file web_material_uhac022.zip › Supplemental Figure.docx]

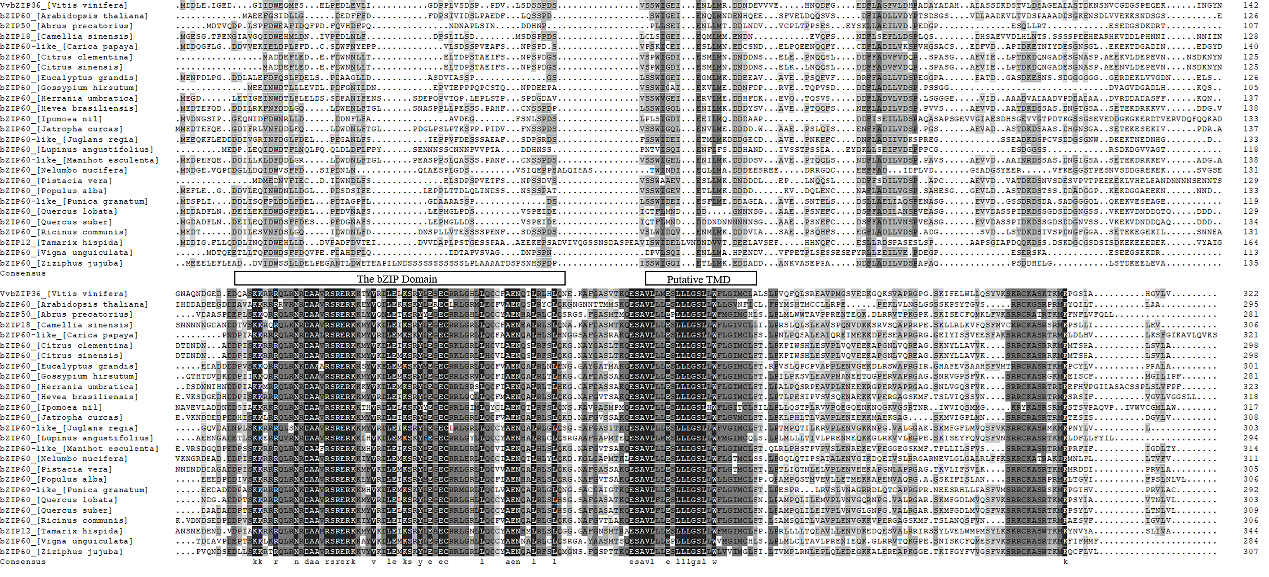


**Fig. S1.** Multiple sequence alignment. Full-length amino acid sequence comparison of VvbZIP36 (VIT_18s0122g00500) with homeologs from other species. *Abrus precatorius* bZIP50 (XP_027366621.1), *Arabidopsis thaliana* bZIP60 (OAP18538.1), *Camellia sinensis* bZIP18 (ALL97705.1), *Carica papaya* bZIP60-like (XP_021909218.1), *Citrus clementina* bZIP60 (XP_006435475.1), *Citrus sinensis* bZIP60 (XP_006473869.1), *Eucalyptus grandis* bZIP60 (XP_010063178.2), *Gossypium hirsutum* bZIP60 (XP_016748430.1), *Herrania umbratica* bZIP60 (XP_021283978.1), *Hevea brasiliensis* bZIP60 (XP_021646411.1), *Ipomoea nil* bZIP60 (XP_019172061.1), *Jatropha curcas* bZIP60 (NP_001292941.1), *Juglans regia* bZIP60-like (XP_018832980.1), *Lupinus angustifolius* bZIP60 (XP_019461939.1), *Manihot esculenta* bZIP60-like (XP_021600128.1), *Nelumbo nucifera* bZIP60 (XP_010270593.1), *Pistacia vera* bZIP60 (XP_031270269.1), *Populus alba* bZIP60 (XP_034931370.1), *Punica granatum* bZIP60-like (XP_031377447.1), *Quercus lobata* bZIP60 (XP_030951078.1), *Quercus suber* bZIP60 (XP_023918417.1), *Ricinus communis* bZIP60 (XP_002510740.1), *Tamarix hispida* bZIP12 (AFO63291.1), *Vigna unguiculata* bZIP60 (XP_027938181.1) and *Ziziphus jujuba* bZIP60 (XP_015880016.1). The conserved bZIP domain and putative trans-membrane domain (TMD) are indicated with black rectangles.


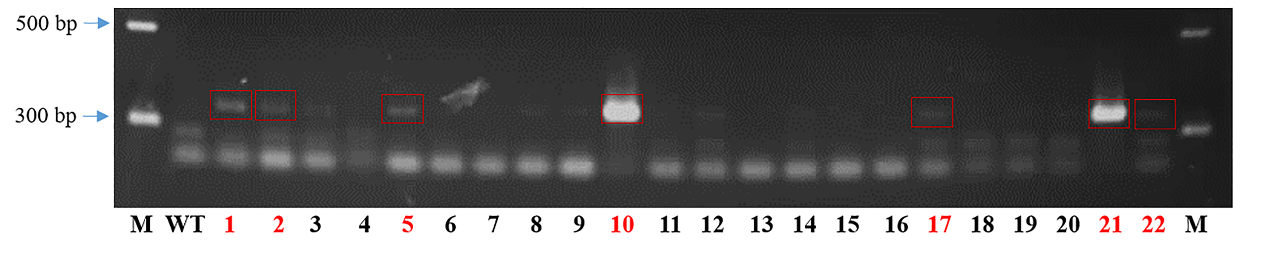


**Fig. S2.** Identification of the T-DNA insertions in 22 regenerated plants. Red numbers and frames represent positive transgenic lines.


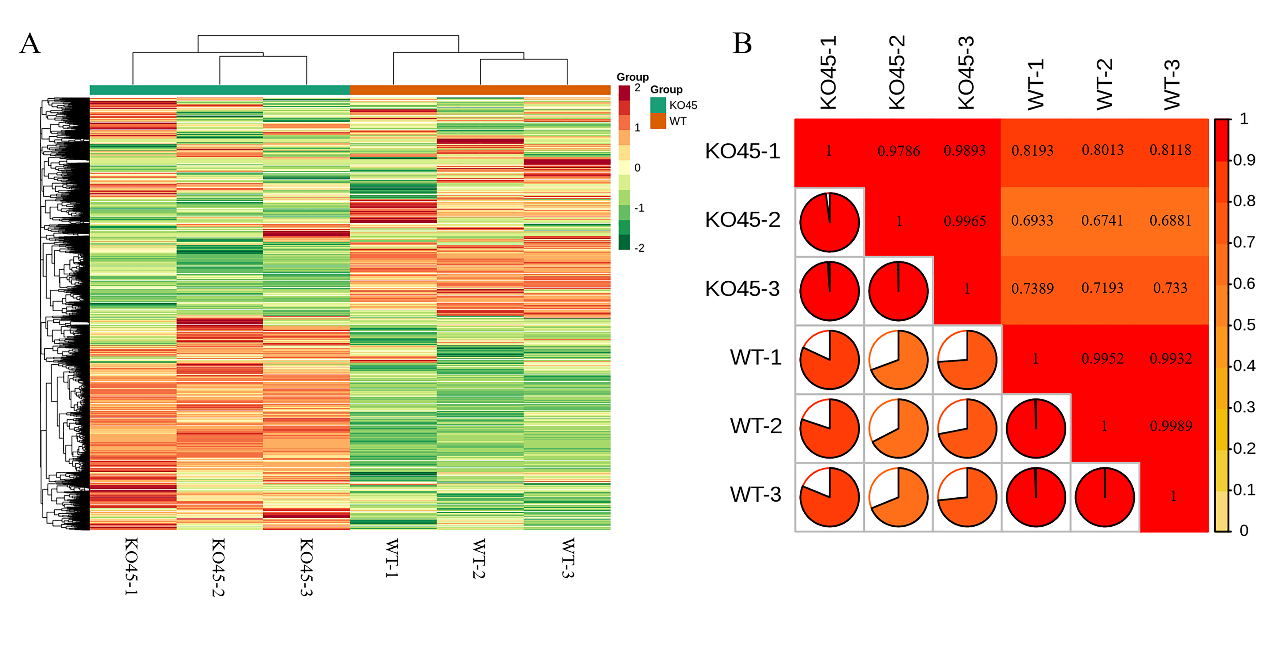


**Fig. S3.** Normalized cluster analysis and Pearson’s correlation analysis of each sample in the transcriptome data.


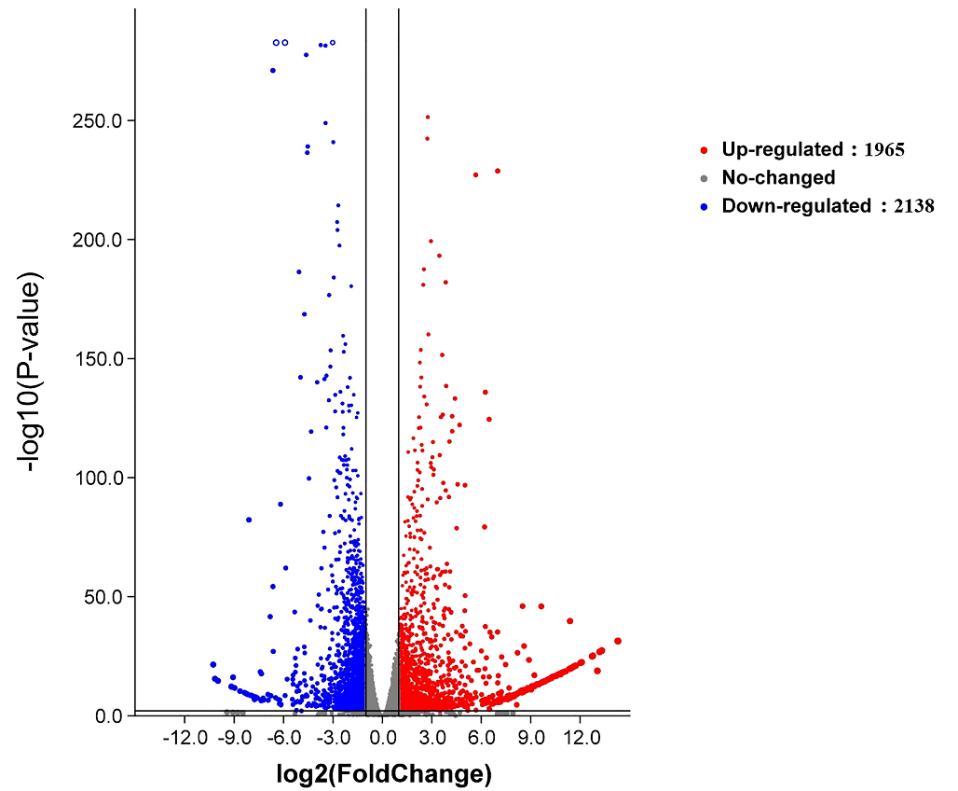


**Fig. S4.** Volcano plot of differentially expressed genes (DEGs). Red dots represent up-regulated genes; green dots represent down-regulated genes; grey dots represent non-differentially expressed genes.


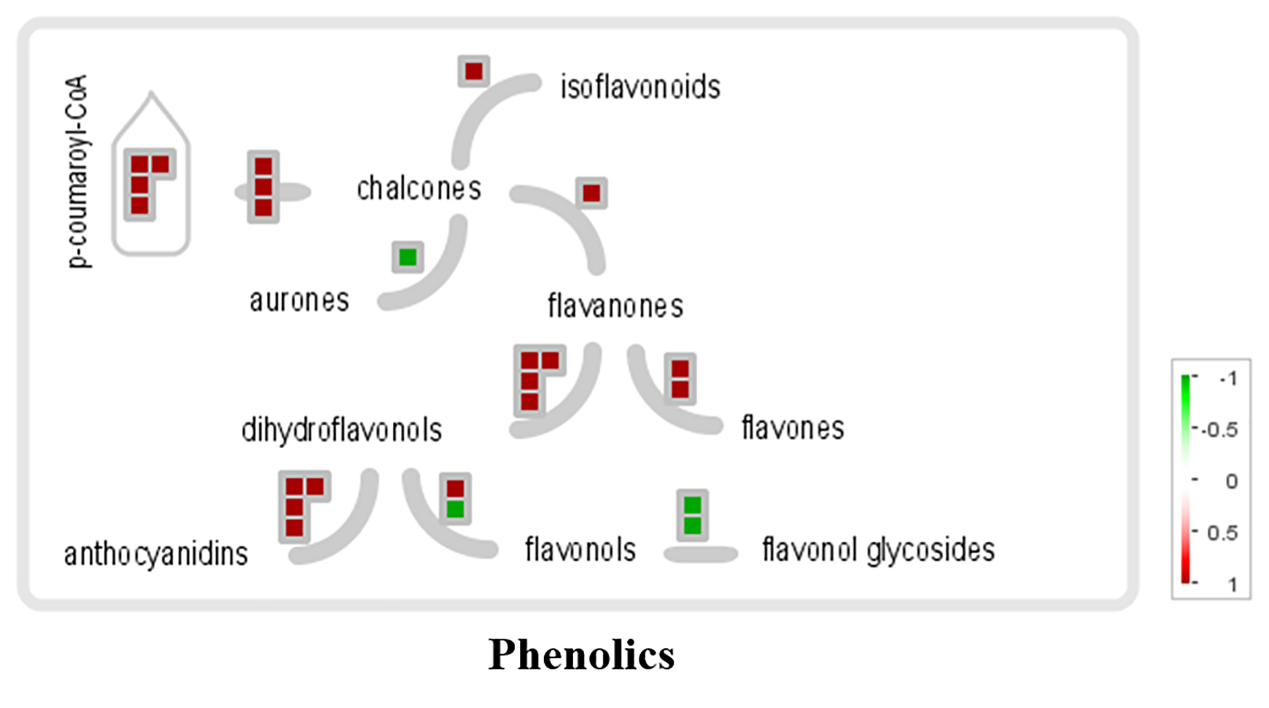


**Fig. S5.** Differentially expressed genes (DEGs) associated with phenolic compound metabolism in KO45, determined by MapMan analysis.


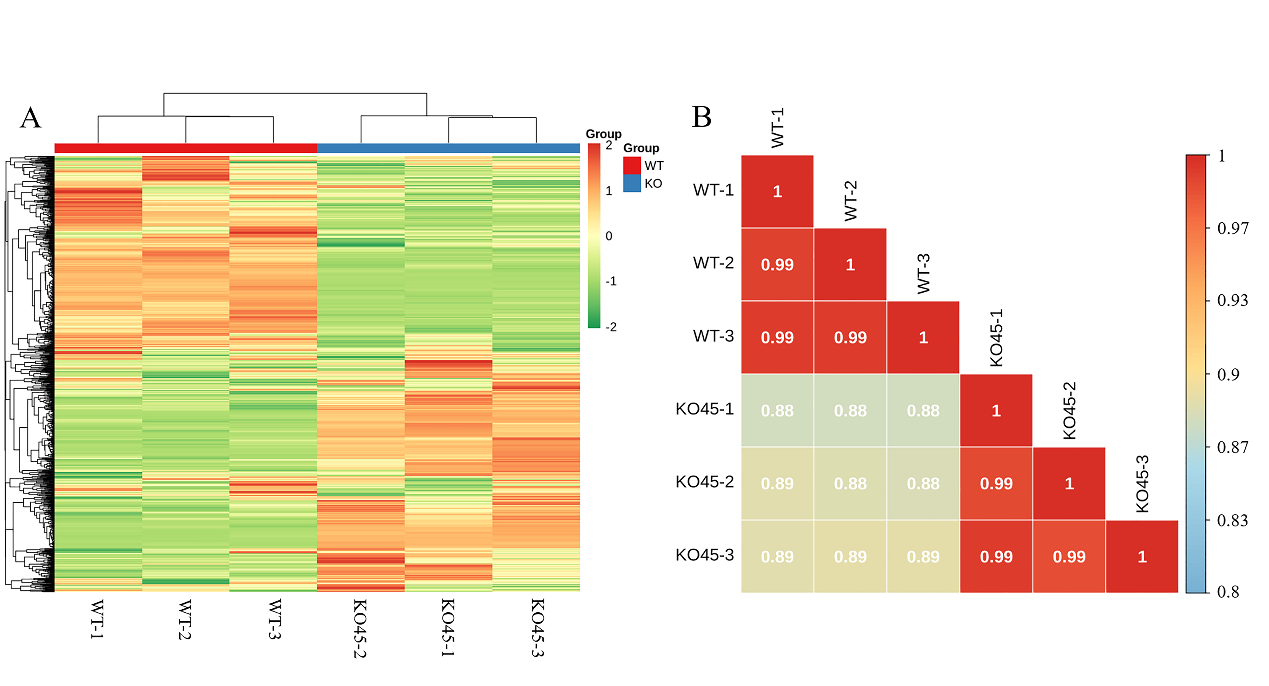


**Fig. S6.** Normalized cluster analysis and Pearson’s correlation analysis of each sample in the metabolome data.


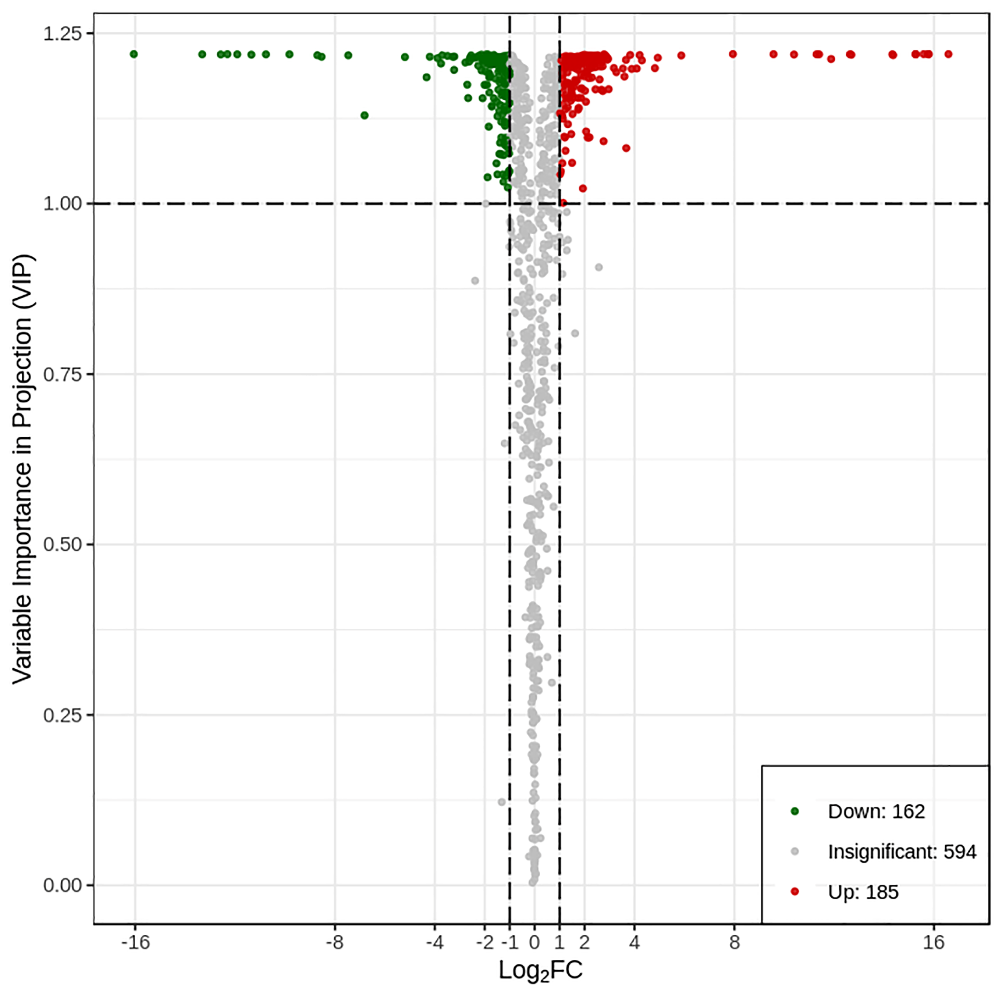


**Fig. S7.** Volcano plot of the differentially abundant metabolites. Red dots represent higher levels of metabolites in the transgenic lines; green dots represent lower levels of metabolites; grey dots represent metabolites that were not present in significantly different amounts.


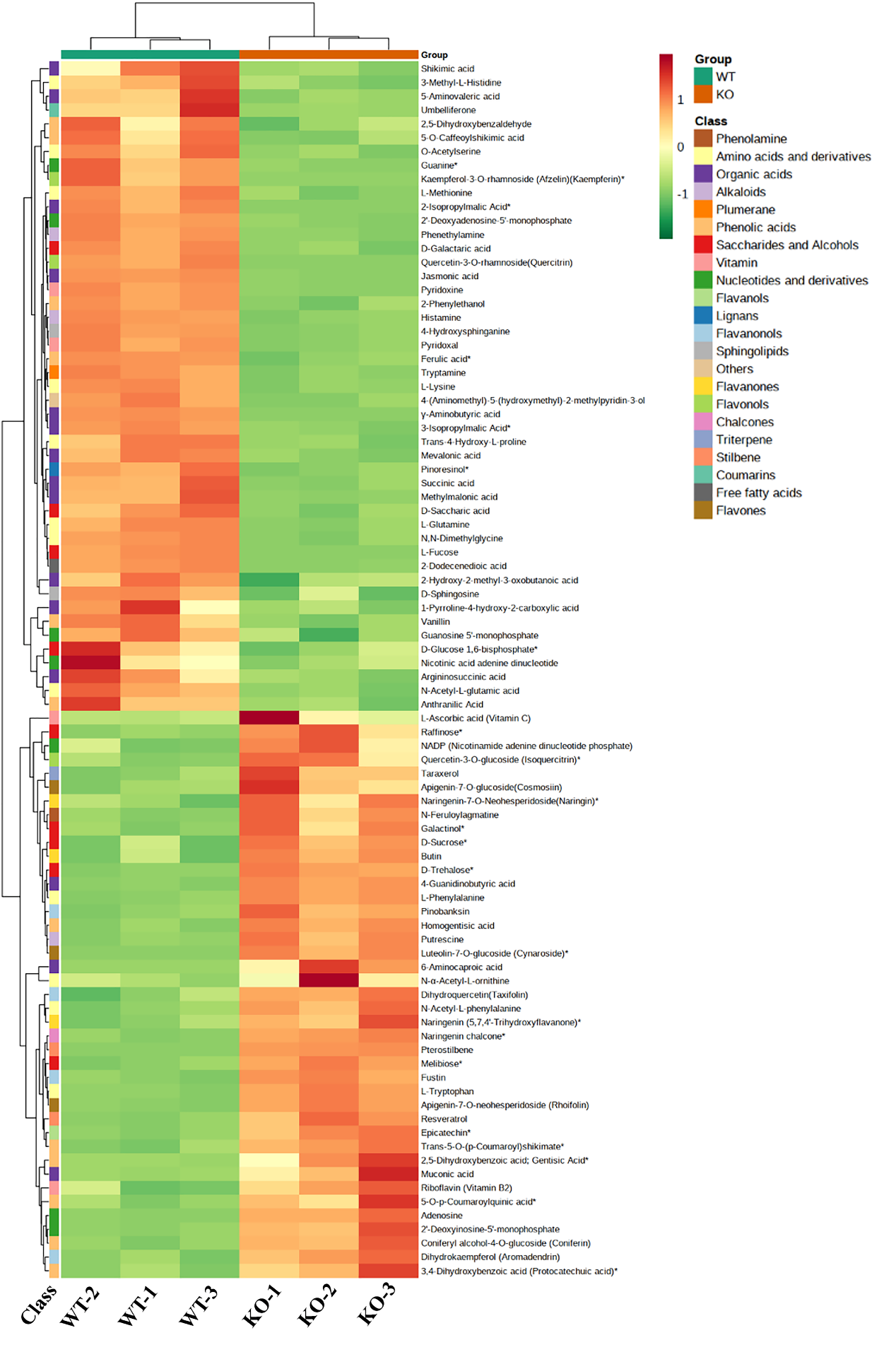


**Fig. S8.** Clustering heat map of differentially abundant metabolites in Kyoto Encyclopedia of Genes and Genomes (KEGG) pathways.


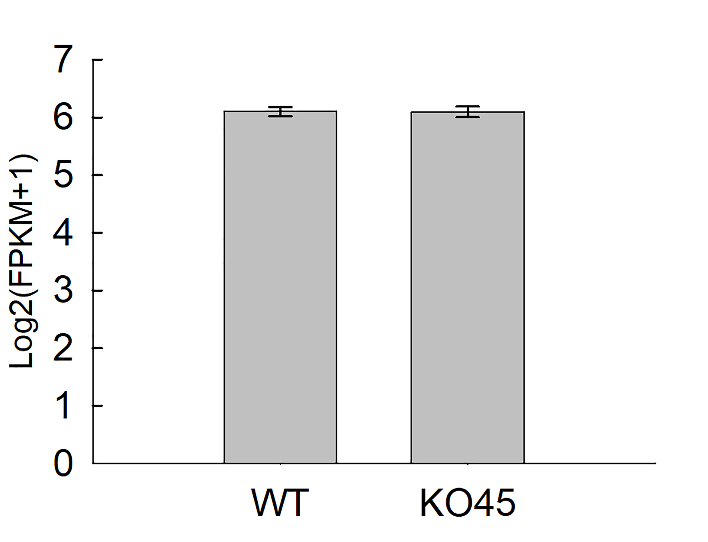


**Fig. S9.** Expression analysis of *VvbZIP36* in KO45 and WT.
