# Supplementary material for: CRISPR/Cas9-mediated mutagenesis of VvbZIP36 promotes anthocyanin accumulation in grapevine (Vitis vinifera)
Source: Hortic Res. 2022 Feb 20;9:uhac022. doi: 10.1093/hr/uhac022 (PMC9174745; doi:10.1093/hr/uhac022)
Supplement: Web_Material_uhac022 [file web_material_uhac022.zip › Supplemental Table S1.docx]

| \| Table S1 Mutation rate of *VvbZIP36* transcript in mutant plant. \| \| \| \| \| \| \| --- \| --- \| --- \| --- \| --- \| --- \| \| Sample \| Wild type \| Mutation \| Mutation rate \| \| ko45-1 \| 342 \| 136 \| 39.77% \| \| ko45-2 \| 381 \| 185 \| 48.56% \| \| ko45-3 \| 248 \| 130 \| 52.42% \| \| Total \| 971 \| 451 \| 46.45% \| |
| --- | --- | --- | --- | --- | --- | --- | --- | --- | --- | --- | --- | --- | --- | --- | --- | --- | --- | --- | --- | --- | --- | --- | --- | --- | --- | --- |

The number represents the reads mapping to the SgRNA1 of *VvbZIP36*.
