# Supplementary material for: CRISPR/Cas9-mediated mutagenesis of VvbZIP36 promotes anthocyanin accumulation in grapevine (Vitis vinifera)
Source: Hortic Res. 2022 Feb 20;9:uhac022. doi: 10.1093/hr/uhac022 (PMC9174745; doi:10.1093/hr/uhac022)
Supplement: Web_Material_uhac022 [file web_material_uhac022.zip › Supplemental Table S2.docx]

| \| Table S2 Specific primers used for qRT-PCR. F, forward; R, reverse. \| \| \| \| \| \| --- \| --- \| --- \| --- \| --- \| \| Gene ID \| Gene names \| Primer sequences (5’-3’) \| \| VIT_16s0039g01100  VIT_05s0136g00260  VIT_13s0067g03820  VIT_18s0001g14310  VIT_17s0000g07210  VIT_01s0011g02960  VIT_18s0001g12800  VIT_02s0025g04720  VIT_16s0098g00860  VIT_13s0047g00210  VIT_15s0045g01490  VIT_09s0002g08090  VIT_03s0091g01080  VIT_18s0001g03430  VIT_06s0009g02010  VIT_04s0044g00580 \| *VvPAL2*  *VvCHS3*  *VvCHI1*  *VvF3H2*  *VvF3'H1*  *VvLAR1*  *VvDFR*  *VvANS*  --  *--*  *VvROMT*  *VvFLS1*  *VvFLS4*  *VvFLS5*  *VvFLR*  *VvActin1* \| F: AAGGTGGAGAGTTTCGTCGT  R: TATCGTAAGCGTTTCACCACCA  F: GAATGAGGCCTTCCAGCCTT  R: TCAAGACGTGTCGTGTGGAG  F: AGAACGTCCTATTTCCGCCG  R: CACTCCGATGGCTGTGAACT  F: GCGAGAAGCTGATGGGTCTT  R: GTTTGAGTCCGAGCGTGAGA  f: AGCACGTTGATCTCGCTGAA  R: TTGGGCAGGTCCAAATCAGTT  f: GAACTCGCATCTGTGTGGGA  R: GTCCTCCGGGCCATCAATAG  F: GGCGAGGAAGCAACAAAACA  R: TGAACCGATGAAACCGGAGG  F: GCCTAAGACACCAAGCGACT  R: ATTCAGGCTGGGGACACTTG  F: CCTCTGGATCAGTACACGCC  R: TCTGTGTGTCCTGGCAATCC  F: CTTAAGGACGGCAAGTGGGT  R: GCTCGGTGCCATACACTTCT  F: AAAAGATGAGCCCCTGACCG  R: CACGCTTGCAATAATCCGGG  F: CAGGCAACAGGTCATGGGAT  R: TCCTTCTTCAGGGGTGGGAT  F: GACAGTTAGGGTTCCGGTGG  R: GTTGGGCTAGTTCTTGGGCT  f: TTGAGAGAGTGCAAGCCCTG  R: GTCGTGAGACTCCGCTAAGG  F: GAGTGCACATCACACATGGC  R: ACACATCCTCTATTGCCCGC  F: GATTCTGGTGATGGTGTGAGT  R: GACAATTTCCCGTTCAGCAGT \| |
| --- | --- | --- | --- | --- | --- | --- | --- | --- | --- | --- | --- |
